# Optimizing Acoustic Performance of Semi-Dense Asphalt Mixtures Through Energy Dissipation Characterization

**DOI:** 10.3390/ma18174086

**Published:** 2025-09-01

**Authors:** Huaqing Lv, Gongfeng Xin, Weiwei Lu, Haihui Duan, Jinping Wang, Yi Yang, Chaoyue Rao, Ruiyao Jiang

**Affiliations:** 1 School of Transportation Engineering, Changsha University of Science & Technology, Changsha 410114, China; hcl20000318@163.com (H.L.); hhduan@csust.edu.cn (H.D.); wangjp@stu.csust.edu.cn (J.W.); 18390079778@stu.csust.edu.cn (C.R.); riojay_1008@163.com (R.J.); 2 Shandong Key Laboratory of Highway Technology and Safety Assessment, Jinan 250000, China; gfxin@163.com; 3 Shandong Hi-Speed Group Co., Ltd., Innovation Research Institute, Jinan 250000, China; 4 Xiandai Investment Co., Ltd., Changsha 410004, China; yangyi@csust.edu.cn

**Keywords:** noise reducing road surface, semi-dense graded asphalt mixtures, energy dissipation, damping ratio

## Abstract

Traffic-induced noise pollution is a significant environmental issue, driving the development of advanced noise-reducing pavement materials. Semi-dense graded asphalt mixtures (SDAMs) present a promising compromise, offering enhanced acoustic properties compared to conventional dense-graded asphalt mixtures while maintaining superior durability to porous asphalt mixtures. However, the mechanism underlying the relationship between the energy dissipation characteristics and noise reduction effects of such mixtures remains unclear, which limits further optimization of their noise reduction performance. This study designed and prepared semi-dense graded noise-reducing asphalt mixtures SMA-6 TM, SMA-10 TM, and SMA-13 TM (SMA TM represents noise-reducing SMA mixture) based on traditional dense-graded asphalt mixtures SMA-6, SMA-10, and SMA-13, and conducted tests for water stability, high-temperature performance (60 °C), and low-temperature performance (−10 °C). Subsequently, energy loss parameters such as loss factor and damping ratio were calculated through dynamic modulus tests to characterize their energy dissipation properties. The mechanism linking the energy dissipation characteristics of semi-dense graded asphalt mixtures to noise reduction was investigated. Finally, the noise reduction effect was further verified through a tire free fall test and a close-proximity (CPX) method. The indoor test results indicate that the semi-dense mixtures exhibited a trade-off in performance: their dynamic stability was 11.1–11.3% lower and low-temperature performance decreased by 4.2% (SMA-13 TM) to 14.1% (SMA-6 TM), with moisture stability remaining comparable. Conversely, they demonstrated superior damping, with consistently higher loss factors and damping ratios. All mixtures reached peak damping at 20 °C, and the loss factor showed a strong positive correlation (R^2^ > 0.91) with energy dissipation. Field results from a test section showed that the optimized SMA-10 TM mixture yielded a significant tire–road noise reduction of 3–5 dB(A) relative to the SMA-13, while concurrently meeting key performance criteria for anti-water ability and durability. This study establishes a link between the energy dissipation in SDAM and their noise reduction efficacy. The findings provide a theoretical framework for optimizing mixture designs and support the wider application of SDAM as a practical noise mitigation solution.

## 1. Introduction

With the acceleration of urbanization, noise pollution caused by vehicle traffic has gradually become a prominent issue on urban roads and surrounding highways [1]. Taking China as an example, transportation noise accounts for as much as 35% of all environmental complaints [2], and traffic-related noise pollution has become a significant detriment to the overall health, both physical and mental, of city inhabitants [3,4,5,6,7,8]. Furthermore, noise pollution imposes significant adverse socio-economic impacts on built-up urban areas, compelling governments and communities to invest substantial additional funds in noise mitigation infrastructure, such as sound barriers [9]. In order to address this issue, numerous researchers have focused on developing low-noise pavement materials that can effectively reduce road surface noise [10,11,12,13,14]. Porous asphalt mixtures (PAMs), with their unique porosity and sound absorption capability, can effectively absorb tire/road noise and demonstrate significant noise reduction effects in real-world road applications [15,16,17]. However, the durability of the pavement and the issue of pore clogging still restrict the further application of PAMs [18,19,20], and, at present, this problem can only be addressed through enhanced maintenance [21,22]. An alternative approach focuses on enhancing material damping, for instance by incorporating crumb rubber, utilizing the excellent damping properties of rubber to absorb vibration-induced noise [23,24]. However, without an optimized porous structure, the overall noise reduction of such dense-graded rubberized mixes often remains limited.

To bridge the gap between the acoustic performance of PAMs and the durability of dense-graded mixtures, semi-dense graded asphalt mixtures (SDAMs) have been developed, which have a denser structure while still retaining some porosity. These pavements offer a combination of sound absorption capability and good structural stability, and have been widely used for low-noise pavement rehabilitation in urban areas and on highways in countries such as Switzerland [25,26,27]. Mikhailenko [13] pointed out that SDAMs exhibit relatively balanced performance in terms of durability, skid resistance, and noise reduction. Steiner et al. [28] found through comparative analysis that the long-term acoustic performance of SDAMs is superior to that of PAMs. Piao et al. [29], from a full life-cycle perspective, also confirmed the stable noise reduction effect of SDAM.

In addition to the basic porous absorption mechanism, recent studies have further revealed that the noise originating from the tire–pavement interaction not only arises from aerodynamic effects but is also influenced by the “mechanical impedance” of the road surface. Its variation can affect the propagation and dissipation of vibrational energy [30]. Compared to PAMs, SDAMs have greater advantages in mechanical impedance characteristics, which can further amplify the damping noise reduction effect [31].

SDAMs exhibit a dual noise reduction mechanism. The noise reduction capability, which is highly dependent on the internal configuration of the mixture, can be improved via purposeful design. On the one hand, their porosity contributes to sound absorption, as demonstrated by Ošernas et al. [31], who measured a sound absorption coefficient as high as 0.53 for an SDAM (BBTM-8). On the other hand, and central to the present study, is the optimization of its mechanical damping performance through gradation design. Research shows a clear trend that finer gradations produce better noise reduction effects. For example, C. Angst [32] demonstrated that SDAM with a smaller nominal maximum aggregate size is more effective in suppressing tire vibration and excitation noise [33,34,35]. This principle was further confirmed in studies on related mixtures, where R. Bernhard et al. [36] observed that a finer SMA-9 mixture significantly reduced noise compared to the coarser SMA-16. This body of work strongly suggests that optimizing gradation design is a key strategy for enhancing the pavement’s energy dissipation capacity while maintaining structural integrity, the latter of which is fundamentally governed by microscopic bitumen–aggregate adhesion, as explored in related material science [37,38].

While many researchers have tested the noise reduction effects of SDAMs, these studies have primarily focused on the final acoustic results. For SDAMs, a systematic relationship between the acoustic energy dissipation characteristic in the asphalt mixture and its measured noise reduction effects has not been established. Although foundational research has begun to forge this connection, these principles have not yet been comprehensively applied to SDAM. For example, V.F. Vázquez et al. [39] proposed a method using the Boltzmann Sigma function to correlate the stiffness spectrum of asphalt mixtures with their acoustic effects. Building upon this, research by K.P. Biligiri et al. [40,41] forged a direct link between the stiffness of a material and the resultant traffic noise, critically demonstrating through stress–strain analysis that lower stiffness enhances energy dissipation, thereby improving noise reduction. Therefore, targeted research that correlates the energy dissipation characteristics of SDAMs with their acoustic performance is crucial for creating a theoretical design framework for future high-performance noise-reducing pavements [41,42].

Therefore, this study focuses on an in-depth investigation of the energy dissipation characteristics of SDAM-type asphalt mixtures on tire/road noise, revealing the mechanisms of noise generation and attenuation within the SDAM-type asphalt mixture system from an energy perspective. To this end, three types of SDAM with different maximum particle sizes were prepared—SMA-6 TM, SMA-10 TM, and SMA-13 TM (where ‘TM’ denotes our specific semi-dense gradation design) [43]—and compared with their corresponding dense-graded asphalt mixtures—SMA-6, SMA-10, and SMA-13. The structural reliability was validated through road performance evaluation, while the energy dissipation mechanism was analyzed by combining dynamic modulus and damping ratio. Additionally, the mechanical damping noise reduction effect was systematically assessed using tire drop tests and CPX noise measurement equipment.

In contrast to previous research on SDMA, which often simply correlated pavement type with noise reduction, this study introduces a novel, mechanism-based framework by establishing a quantitative link between viscoelastic energy dissipation and acoustic performance. The validation through both lab and field tests confirms that our approach not only enables the predictable creation of quieter pavements but also effectively resolves the long-standing conflict between acoustic performance and durability, thus offering a more scientific and efficient pathway to mitigating traffic noise pollution.

## 2. Materials and Methods

### 2.1. Raw Material

#### 2.1.1. Aggregate

The aggregates used in this study were basalt, with particle size ranges primarily of 0–5 mm and 5–10 mm, and partially 10–15 mm for certain gradations. The mineral filler was limestone. The properties of the aggregates, summarized in Table 1, were determined following the standard *Test Methods of Aggregate for Highway Engineering (JTG E42-2005)* [44].

#### 2.1.2. Asphalt

This study used high viscosity asphalt produced by Changsha University of Science and Technology, designated as PG94-10. The conventional properties of this asphalt were tested, the results of which are detailed in Table 2.

#### 2.1.3. Additive

Lignin fiber and P.C 42.5 cement were introduced as additives during the mixture preparation. The former, characterized by a density of 2.482 g/cm^3^ and a maximum length of 6 mm, constituted 0.3% of the total mixture mass. The latter was added at a rate of 1% by mass of the aggregate.

**Figure 1:**
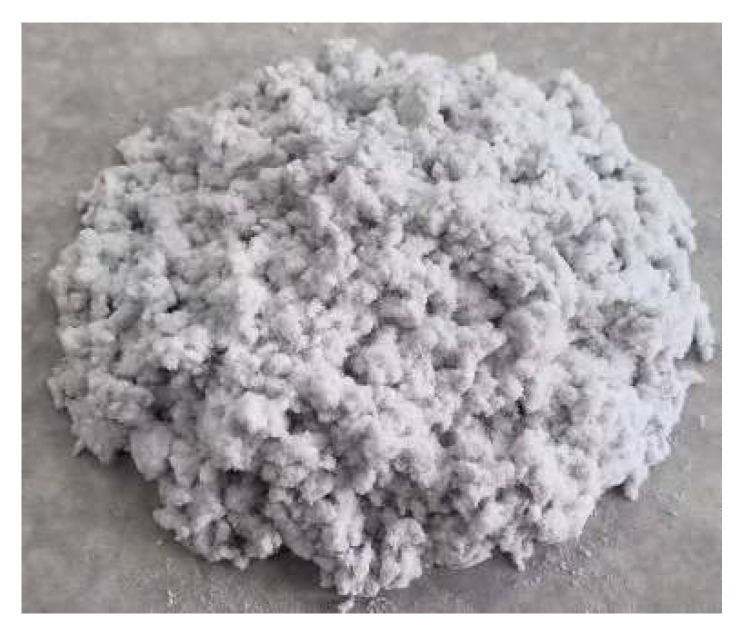
Additive—Lignin Fiber.

The fiber stabilizer to be added shall be high-quality lignin flocculent fiber. The fiber shall not deteriorate or become brittle at a dry mixing temperature of 250 °C, and must meet environmental protection requirements without endangering human health. The lignin fiber used in this study is provided by Guangdong Lantai Environmental Protection Technology Co., Ltd., (Dongguan, China) with specific parameters shown in Table 3, and lignin fibers are shown in Figure 1.

### 2.2. Mix Design

**Figure 2:**
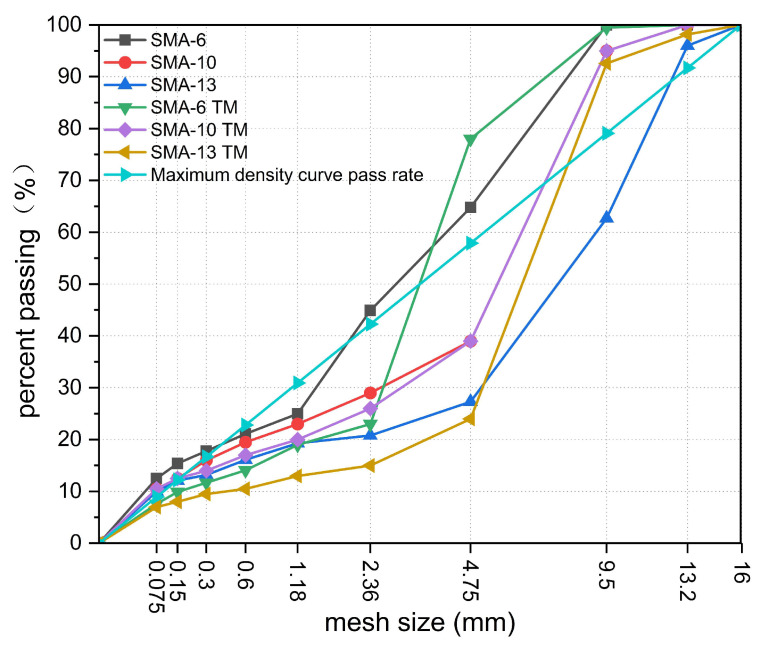
Grading curve.

To investigate the noise reduction effect of SDAMs, this study is based on SMA-6, SMA-10, and SMA-13. Drawing on the relevant research results by Lin and others in Hong Kong [46], the Marshall design method was employed. By optimizing parameters such as asphalt consistency, asphalt content, aggregate particle size, and mix proportion, the void content of the mixture was controlled between 9% and 11%, resulting in three semi-dense graded mix designs: SMA-6 TM, SMA-10 TM, and SMA-13 TM. Conventional dense-graded SMA-6, SMA-10, and SMA-13 were used as control groups to systematically study their noise reduction effects and mechanisms. The gradation curves of the six mixtures are shown in Figure 2.

Control over the asphalt-to-aggregate ratio and void content was strictly maintained in accordance with the *Specification for Tests of Asphalt and Asphalt Mixtures for Highway Engineering (JTG E20-2011)* and international engineering practices. This approach was adopted to isolate the effect of the maximum nominal particle size on the mixture’s noise reduction performance by minimizing interference from other material factors, such as asphalt content variations. A series of specimens were prepared at varying binder contents, and the optimum asphalt content (OAC) was selected as the content that achieved the target air void content while meeting other critical Marshall criteria. The optimal asphalt–aggregate ration for the six mixtures was determined through preliminary tests, as shown in Table 4.

### 2.3. Pavement Performance

#### 2.3.1. Water Stability

Based on the JTG E20-2011 (T0729-2000) standard, we evaluated the water stability of asphalt mixtures through immersion Marshall tests and freeze–thaw splitting tests, verifying their ability to resist moisture-induced damage under high-temperature water bath and freeze–thaw cyclic conditions. The immersion Marshall test compares the Marshall stability values MS_0_ and MS_1_ of specimens treated in a water bath at room temperature for 30 min and at 60 °C for 48 h, respectively, to calculate the Immersion Residual Stability (IRS). The freeze–thaw splitting test involves conducting splitting tests after freeze–thaw cycles and calculating the Tensile Strength Ratio (TSR).

#### 2.3.2. High-Temperature Performance

The wheel-tracking test was employed to assess the high-temperature performance, yielding the dynamic stability (DS) value. This index reflects the material’s ability to resist deformation and maintain structural stability under repeated high-temperature loading. Better high-temperature stability of the material is reflected by a higher dynamic stability (DS) value. Rutting specimens with dimensions of 300 × 300 × 50 mm were prepared using a wheel-tracking molding mechanism, with the subsequent rutting test performed at 60 °C. The entire testing process and the calculation method for DS are in accordance with the JTG E20-2011 (T0719-2011) standard.

#### 2.3.3. Low-Temperature Performance

The low-temperature performance of the mixture is evaluated through a low-temperature bending test. In the test, the maximum flexural strain of the mixture beam is recorded to characterize its low-temperature performance. Superior low-temperature cracking resistance of the mixture is characterized by a larger failure strain. The test specimens are beams with dimensions of 250 × 30 × 35 mm, cut from standard rutting plates. The test was conducted at a temperature of −10 °C with a constant loading rate of 50 mm/min. The testing method follows the standard JTG E20-2011 (T0715-2011).

### 2.4. Viscoelastic Characterization and Energy Dissipation Analysis

#### 2.4.1. Dynamic Modulus Test

Uniaxial compression dynamic modulus tests were performed on an MTS-809 universal testing machine, following the JTG E20-2011 (T0738-2011) standard, which yielded the dynamic modulus and phase angle of the mixture. The loading protocol was executed under unconfined conditions, employing a test matrix that combined five temperatures (−10, 5, 20, 35, and 50 °C) with six loading frequencies (25, 10, 5, 1, 0.5, and 0.1 Hz). During the test, three high-precision displacement sensors are fixed at equal intervals on the surface of the specimen to measure the compressive deformation of the specimen. After loading, the axial stress amplitude σ_0_ and axial strain amplitude Ɛ_0_ at different frequencies are recorded, from which the dynamic modulus |E*| and phase angle *φ* are calculated. The calculation formulas are shown in Equations (1) and (2).

(1) $$\left|E*\right|=\frac{\sigma_{0}}{{Ɛ}_{0}}$$

(2) $$\varphi =\frac{t_{i}}{T}\times 360$$

**Figure 3:**
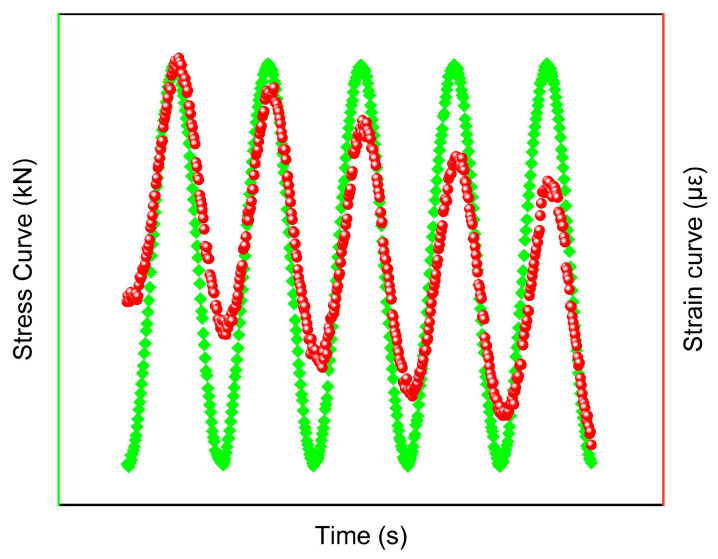
Schematic diagram of dynamic modulus test waveform.

In the equations, *T* is a specimen with one cycle, *t_i_* is the lag time (s), and the parameters refer to Figure 3.

#### 2.4.2. Master Curve Construction

As typical viscoelastic materials, asphalt mixtures exhibit a stress–strain behavior that is highly dependent on temperature, loading conditions, and time. This dependency is fundamentally governed by the time–temperature equivalence principle [47]. The master curve of the dynamic modulus is typically described using a Sigmoidal model [48], as shown in Equations (3)–(5):

(3) $$log\left|E*\right|=\delta +\frac{\alpha }{1+\exp\left(\beta +\gamma \log f_{r}\right)}$$

(4) $$\log\left(f_{r}\right)=\log\left(f\right)+\log\left[\alpha \left(T\right)\right]$$

(5) $$\alpha \left(T\right)=\exp\left(-b\left(T-T_{0}\right)\right)$$

In the equations, $f_{r}$ represents the reduced frequency at the reference temperature (Hz); δ represents the minimum value of dynamic modulus; α represents the difference between the maximum and minimum values of the dynamic modulus; β and γ, respectively, represent the shape parameters of the main curve of dynamic modulus. f represents the loading frequency at 20 °C (Hz); $\alpha \left(T\right)$ represents the shift factor, and b is the regression parameter of the model; and T and $T_{0}$ are the tests temperature and reference temperature, respectively, in °C.

Construction of the phase angle master curve was performed by applying the differential form of the Sigmoidal function. This process utilized a reference temperature of 20 °C, and the WLF equation was employed to determine the displacement factors [49]. The model parameters of the phase angle master curve are fitted using the least squares method, as shown in Equation (6).

(6) $$\varphi =90\times \frac{dlog\left|E*\right|}{\mathrm{dlog}f_{r}}=90\times \frac{-k\alpha \gamma \times \exp\left(\beta +\gamma \log f_{r}\right)}{{\left[1+\exp\left(\beta +\gamma \log f_{r}\right)\right]}^{2}}$$

In Equation (6), all parameters except for k are shared with the main curve of the dynamic modulus, where k is an auxiliary fitting parameter for the phase angle main curve.

Finally, utilizing the experimentally obtained dynamic modulus and phase angle data, the dynamic modulus–phase angle curve is plotted and fitted using the Lorentzian asymmetric curve *L(x)*, as shown in Equations (7) and (8).

(7) $$L\left(x\right)=\frac{1}{1+{4\left(\frac{x-x_{c}}{w}\right)}^{2}}$$

(8) $$y=y_{0}+\frac{2A}{\pi w}\left\{\begin{array}{c} {\left(L\left(x\right)\right)}^{p_{1}},x\leq x_{c}\\ {\left(L\left(x\right)\right)}^{p_{2}},x>x_{c} \end{array}\right.$$

In Equations (7) and (8), $x_{c}$ represents the dynamic modulus corresponding to the peak value, $y_{0}$ is the asymptotic value of y when x deviates from $x_{c}$, A is the peak amplitude parameter, w is the peak width parameter, and $p_{1}$, $p_{2}$ control the decay exponents on the left and right sides of the peak, respectively. Based on the given formulas, it can be observed that the larger the peak determined by A, the larger the corresponding phase angle. Additionally, as w increases, the dynamic modulus range covered by the curve broadens, while $p_{1}$ and $p_{2}$ determine the rate at which the phase angle increases or decreases with the dynamic modulus.

#### 2.4.3. Energy Dissipation Parameters

The dynamic modulus is expressed in complex form, comprising the storage modulus E′ and the loss modulus E″. The storage modulus E′ represents the material’s ability to store elastic energy, characterizing its elastic behavior. The loss modulus E″ represents the material’s ability to dissipate energy, characterizing its viscous behavior. Both the storage modulus E′ and the loss modulus E″ can be calculated through uniaxial compression dynamic modulus tests, as given in Equation (9):

(9) $$E^{\prime }=\frac{\sigma_{0}}{{Ɛ}_{0}}cos\varphi ,E^{\prime \prime }=\frac{\sigma_{0}}{{Ɛ}_{0}}sin\varphi$$

where $\sigma_{0}$, ${Ɛ}_{0}$, and φ represent the stress amplitude, strain amplitude, and phase angle, respectively.

Due to the variations in specimen size, the energy loss of the material during the dynamic loading process also differs, which makes it impossible to compare mixtures with different gradations by directly calculating the total energy loss. Therefore, the energy dissipation capacity for different gradations is evaluated by calculating the energy loss factor η and the energy loss per unit volume W_L_, as shown in Equations (10) and (11).

(10) $$\eta =\frac{E^{\prime \prime }}{E^{\prime }}=tan\varphi$$

(11) $$W_{L}=\frac{1}{2}E\prime \prime \varepsilon_{0}$$

### 2.5. Damping Analysis

#### 2.5.1. Damping Ratio

When a vehicle is in motion, the vibration at the tire–pavement interface is gradually attenuated by damping forces. A portion of this energy is dissipated as thermal energy, while another part is transformed into acoustic noise. Both of these are forms of energy loss for the system. With a greater portion of vibrational energy undergoing conversion to thermal energy, the generated noise is correspondingly reduced. The conversion process of this vibrational energy directly affects the manner of energy dissipation and the resulting noise level.

**Figure 4:**
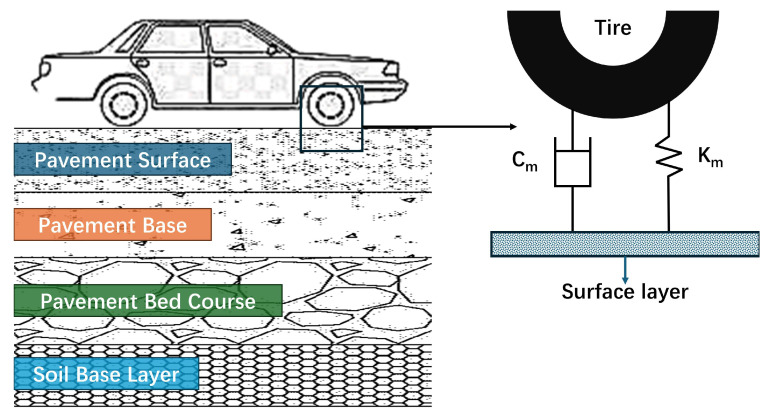
Schematic diagram of tire–road damping system.

The vibration transmission between the vehicle tire and the pavement surface can be simplified as a component consisting of a spring with an elastic coefficient K_m_ and a damper with a damping coefficient C_m_ in parallel. During vehicle operation, the tire acts as the driving system and the pavement as the energy storage system. Resonance may occur when energy is transferred between these two systems, as shown in Figure 4. The damping ratio and angular natural frequency of this model can be calculated using Equations (12) and (13).

(12) $$\zeta =\frac{R_{m}}{2M_{m}\omega_{0}}$$

(13) $$\omega_{0}=2\pi f_{0}=\sqrt{K_{m}/M_{m}}$$

where ζ is the damping ratio; $\omega_{0}$ is the angular natural frequency, also known as the undamped natural frequency; $R_{m}$ is the resistance coefficient; and $M_{m}$ represents the relevant tire/axle assembly.

When a vehicle passes over the pavement, the excitation force generated by the tire is transmitted to the pavement, causing it to undergo simple harmonic vibration. The relationship of the tangent of the phase angle, tan(φ), with the material damping coefficient $C_{m}$ and stiffness $K_{m}$ is shown in Equation (14) [41]:

(14) $$\tan\left(\varphi \right)=\frac{C_{m}}{K_{m}}$$

Bert et al. [50] defined the relationship between the material’s critical damping coefficient *C_m,c_*, its stiffness *K_m_*, mass *M_m_*, and natural angular frequency $\omega_{0}$, as shown in Equation (15). By combining the aforementioned equations, under the resonance condition ($\omega_{d}\;$=${\;\omega }_{0}$), the formula for the damping ratio is shown in Equation (16):

(15) $${\left(\frac{C_{m,c}}{2M_{m}\omega_{0}}\right)}^{2}=K_{m}/M_{m}={\omega_{0}}^{2}$$

(16) $$\zeta =\frac{tan\varphi }{2}$$

#### 2.5.2. Energy Amplification Coefficient and Transfer Coefficient

The attenuation pattern of sound waves generated by a tire rolling on the pavement and propagating along a continuous channel is analogous to simple harmonic vibration. The transmission ratio under a static load F_0_, described by the dynamic amplification factor (${DMF}_{max}$), reflects the degree of acoustic energy attenuation, as shown in Equations (17) and (18).

(17) $$x\left(t\right)=\frac{F_{0}}{K_{m}}DMFcos\left(\omega_{0}+\varphi \right)$$

(18) $${DMF}_{max}=\frac{1}{2\zeta }$$

where $F_{0}$ is the amplitude of the load vibration, and $\omega_{0}$ is the angular frequency of the applied load.

Assuming the amplitude of the excitation force is X_0_ and the amplitude of the force transmitted to the vibration system is X_1_, their ratio, *T*, is called the transmission coefficient, as shown in Equation (19).

(19) $$T=\sqrt{1+4\zeta^{2}}=\sqrt{1+{DMF}^{2}}$$

### 2.6. Noise Testing

#### 2.6.1. Tire Free Fall Test

We evaluate the damping and noise reduction effect of the mixture using an improved version of the tire free fall test [51]. This test involves releasing an automobile tire vertically from a certain height, causing it to collide with the pavement surface, and recording the noise level during the collision. To simulate actual road noise conditions as closely as possible, this test used the standard trailer tire supplied with the CPX equipment. Noise measurement was performed using a BSWA-308 sound level meter, produced and manufactured by Beijing Shengwang Sound and Electric Technology Co., Ltd. in Beijing, China, which can automatically collect and analyze sound data. Fabrication of the rutting slabs adhered to the procedures outlined in the JTG E20-2011 standard.

**Figure 5:**
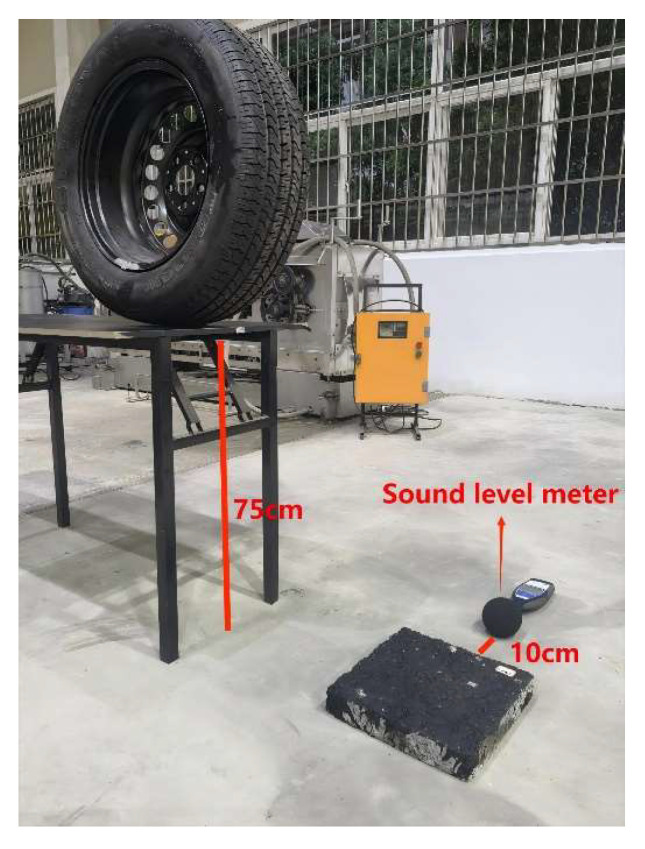
Tire drop experiment.

Through multiple preliminary tests, it was determined that when the tire was dropped from a height of 75 cm, the noise level was between 88 and 95 dB(A), which is consistent with the average noise level measured by the CPX equipment on a newly paved road surface at 85 km/h. Therefore, 75 cm was uniformly adopted as the standard drop height for subsequent tests. The tests procedure is shown in Figure 5; the sound level meter was positioned 10 cm from the rutting slab. The rutting slabs were conditioned at various temperatures for 5 h to ensure a uniform temperature between the interior and the surface, and the test was conducted immediately after the conditioning was complete.

#### 2.6.2. Actual Road Noise Testing

To assess the noise abatement performance of the semi-dense graded asphalt mixture under real-world engineering conditions, SMA-10 TM and SMA-13 trial sections were paved on 29 December 2024, on the Changsha Lituo underpass section of the G4 Beijing–Hong Kong–Macau Expressway. The constituent materials for the trial sections were the same as those investigated in the laboratory phase of this research, with an actual air void content of 9.2%.

**Figure 6:**
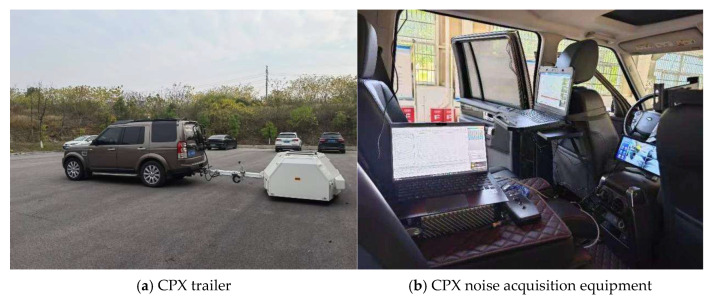
CPX trailer and sensor acquisition device.

Noise testing was conducted in accordance with the test method for Close-Proximity Measurement of Tire/Pavement Noise Influence (JT/T 1465-2023) [52], using a Close Proximity (CPX) measurement system for road noise, as shown in Figure 6. During the tests, the CPX trailer collected noise data while traveling at speeds of 80 km/h and 100 km/h, respectively. To avoid interference from surrounding vehicles and ambient temperature, the test section was completely closed to traffic before testing, and the measurements were performed after the mixture had cooled to ambient temperature. The noise reduction effect of the semi-dense graded asphalt mixture was evaluated based on the collected noise data. It is important to note that the final noise levels and dB(A) reductions reported in this study were calculated as a weighted average, in accordance with the JTT 1465-2023 standard. This procedure accounts for a realistic, mixed traffic composition, thereby incorporating the acoustic influence of both light passenger cars and heavy-duty vehicles to ensure the results reflect practical, on-road conditions.

## 3. Results and Discussion

### 3.1. Pavement Performance

#### 3.1.1. Water Stability Performance

**Figure 7:**
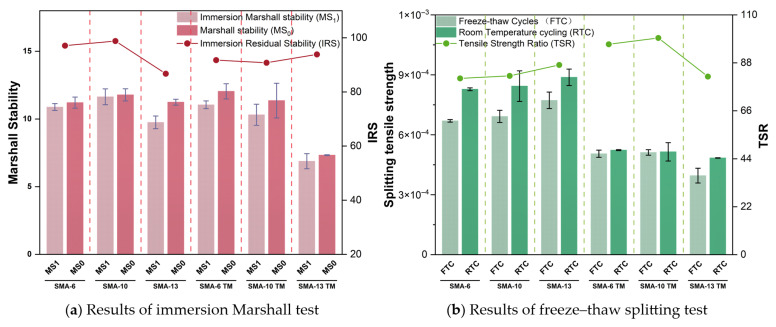
Test results of water stability.

Moisture stability reflects the resistance of an asphalt mixture to moisture-induced damage, and both the TSR and IRS values are closely related to this capacity, as shown in Figure 7.

As shown in Figure 7a, the high-temperature water bath has a minor effect on the retained stability of the asphalt mixtures; only the SMA-13 TM shows a slight decrease due to its larger porosity and maximum nominal particle size. Overall, the semi-dense graded mixtures exhibit higher moisture stability at high temperatures. The results of the freeze–thaw splitting test, shown in Figure 7b, indicate that freeze–thaw cycles have a significant effect on the semi-dense graded mixtures, causing a significant decrease in their splitting strength, although their tensile strength is increased compared to the SMA. The difference in moisture stability between the semi-dense and dense-graded mixtures is primarily determined by porosity, especially during freeze–thaw cycles where water freezes into ice crystals and damages the aggregate skeleton; the larger the porosity, the more pronounced the effect.

#### 3.1.2. High-Temperature Performance

**Figure 8:**
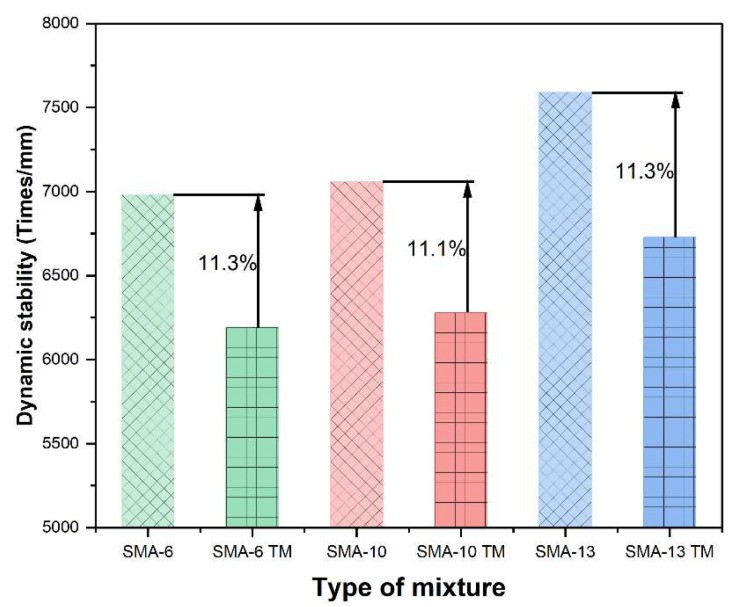
Dynamic stability of asphalt mixture.

The high-temperature performance of an asphalt mixture is defined as its capacity to withstand repeated loading at elevated temperatures while resisting permanent deformation, such as rutting. An asphalt mixture with good high-temperature performance can withstand significant traffic loads under high-temperature conditions and maintain pavement stability. Figure 8 presents the results of the high-temperature performance test.

As the maximum nominal aggregate size increases, the dynamic stability of both the semi-dense graded SMA TM and the dense-graded SMA mixtures improves; the larger the aggregate size, the better the high-temperature performance. However, the higher porosity of the semi-dense graded mixtures causes a slight decrease in their high-temperature performance. Specifically, the dynamic stabilities of SMA-6 TM, SMA-10 TM, and SMA-13 TM decreased by 11.3%, 11.1%, and 11.3%, respectively, compared to the dense-graded mixtures with the same aggregate sizes. Despite these differences, the DS of all six asphalt mixtures meets the specification requirements.

Under high-temperature conditions, asphalt viscosity decreases, and the shear strength is predominantly governed by internal friction resistance [53]. Increasing the aggregate size helps to increase the proportion of coarse aggregate, which enhances internal friction and increases the stability of the aggregate skeleton [54,55]. However, the higher porosity of SMA TM reduces aggregate contact, leading to lower shear strength and consequently affecting high-temperature stability [56].

#### 3.1.3. Low-Temperature Performance

**Figure 9:**
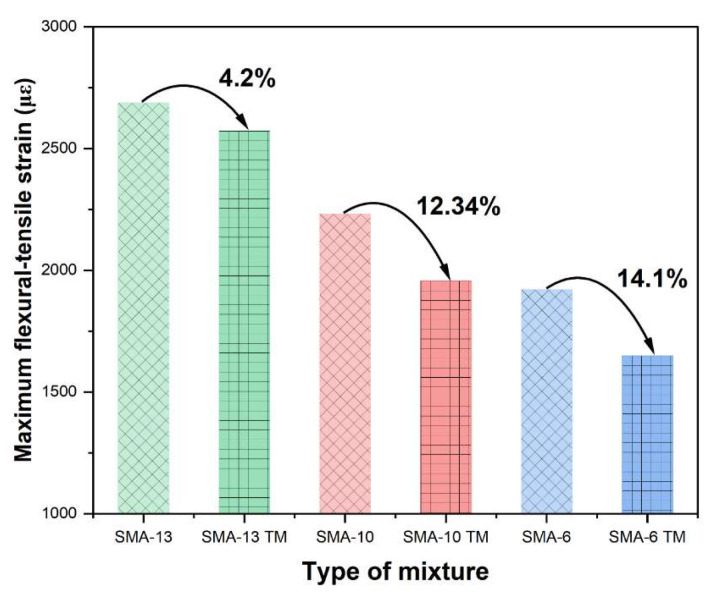
Results of low-temperature splitting test.

The low-temperature performance of an asphalt mixture, which describes its resistance to brittle fracture in cold conditions, is generally evaluated based on its flexural strain at low temperatures. A greater failure strain indicates a stronger resistance to low-temperature cracking. Figure 9 illustrates the outcomes of the low-temperature performance evaluation.

As the maximum aggregate size decreases, the low-temperature flexural strain of the asphalt mixture decreases, and its low-temperature performance deteriorates. Furthermore, the low-temperature performance of SMA-13 TM decreased by 4.2% compared to SMA-13, while that of SMA-6 TM decreased by 14.1% compared to SMA-6. The data indicate that an increase in porosity is detrimental to the low-temperature performance, with the negative impact of porosity being particularly exacerbated in mixtures containing smaller maximum. The low-temperature performance of all six gradations meets the specification requirements.

A larger maximum aggregate size can enhance the contact forces between aggregates and improve low-temperature cracking resistance because the contact forces are more uniformly distributed with larger aggregates, which helps to reduce crack generation. However, due to their higher porosity, semi-dense graded mixtures have looser aggregate contact, which can easily lead to stress concentration and thereby reduce low-temperature performance, an effect that is particularly significant with smaller aggregate sizes [57,58].

### 3.2. Energy Dissipation Analysis

#### 3.2.1. Main Curve Analysis

**Figure 10:**
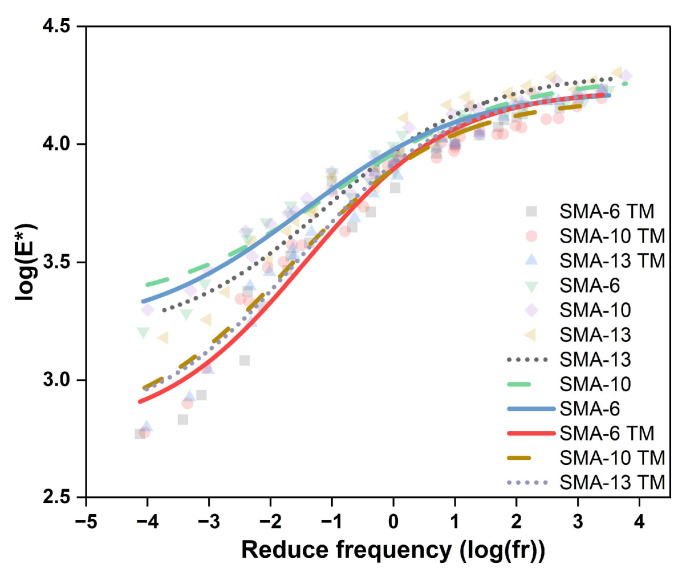
Comparison chart of dynamic modulus main curves.

Figure 10 presents the master curves for the dynamic modulus of the tested mixtures.

Within these curves, the reduced frequency scale is inversely related to temperature, meaning high frequencies correspond to low temperatures and vice versa. The fitting results show that all correlation coefficients are greater than 0.95, indicating a high degree of consistency between the calculated and measured values. The dynamic modulus of the asphalt mixtures increases as the reduced frequency increases. The analysis in Figure 9 indicates that for the dense-graded asphalt mixtures, the dynamic modulus at low temperatures increases as the nominal maximum aggregate size increases, whereas at high temperatures, the dynamic modulus of the SMA-13 mixture decreases. In contrast, the dynamic modulus of the semi-dense graded asphalt mixtures changes less and is less affected by the nominal maximum aggregate size. Notably, at a reduced frequency of zero, the fitted dynamic modulus curves of SMA and SMA TM overlap, indicating that the influence of aggregate size on the dynamic modulus approaches zero. For asphalt mixtures with the same nominal maximum aggregate size, the dynamic modulus reduces as porosity increases, but this difference gradually decreases as the reduced frequency increases.

From the perspective of energy dissipation, the variation in the dynamic modulus of an asphalt mixture is closely related to the material’s energy dissipation behavior. At low temperatures and high frequencies, the material exhibits a high dynamic modulus, corresponding to greater stiffness and minimal energy dissipation. In contrast, at high temperatures and low frequencies, the dynamic modulus is substantially lower, reflecting reduced stiffness and a significant increase in energy dissipation. The dynamic modulus of SMA TM is consistently lower than that of SMA, and its energy dissipation characteristics are relatively stable and at a higher level. When the reduced frequency is zero, the sensitivity of the dynamic modulus to the nominal maximum aggregate size disappears, and energy dissipation is primarily determined by the material’s viscoelastic equilibrium state.

**Figure 11:**
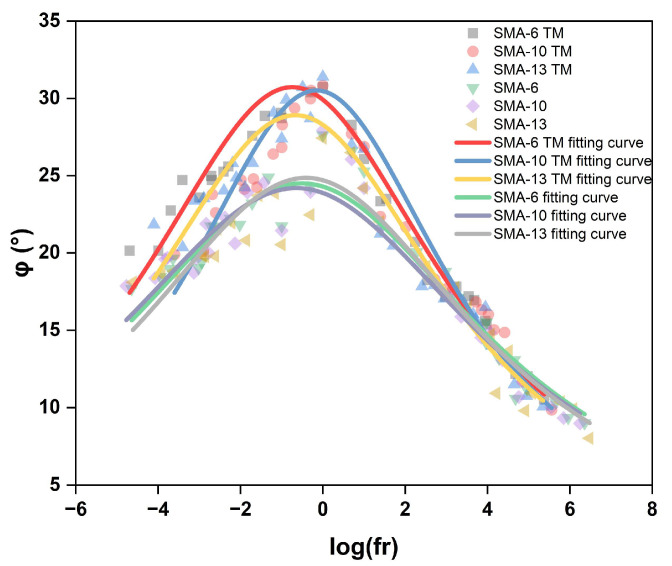
Phase angle main curve.

The viscoelastic balance of the material can be analyzed through the phase angle master curve, as shown in Figure 11. The correlation coefficients are all greater than 87%, indicating that the model can effectively predict the variation in the phase angle with the reduced frequency.

Figure 11 shows that as the reduced frequency increases, the phase angle for the different graded asphalt mixtures first increases and then decreases, and the phase angles for the semi-open graded asphalt mixtures are markedly larger. At low reduced frequencies, the difference in the phase angles of the mixtures is large, but as the frequency increases, this difference gradually diminishes. This indicates that under low-temperature, high-frequency loading, the asphalt mixture exhibits elastic properties, whereas as the loading frequency decreases and temperature increases, its viscous fluid properties become more prominent, and the strain lag is intensified. The phase angle peaks at a reduced frequency between −1 and 0. For the SMA TM, as the nominal maximum aggregate size increases, the phase angle slightly decreases; conversely, the fitted curves for the dense-graded SMA mixtures show that at low air void contents, the influence of aggregate size on the phase angle is very weak.

An analysis combining the phase angle and dynamic modulus master curves reveals that while the difference in dynamic modulus between the two mixture types is small, the phase angle of the semi-open graded mixture is significantly higher than that of the dense-graded mixture, leading to a higher proportion of loss modulus and stronger energy dissipation capacity, thus exhibiting a better noise reduction effect. Under high-temperature conditions, the gap between their phase angles narrows, causing the noise reduction effect of the semi-open graded mixture to decrease slightly. In contrast, under low-temperature conditions, the dynamic modulus and phase angles of the two mixtures tend to converge, their energy dissipation capacities become similar, and, consequently, the noise reduction effect is smaller, relying mainly on sound absorption by the pores.

#### 3.2.2. Correlation Analysis Between Dynamic Modulus and Phase Angle

**Figure 12:**
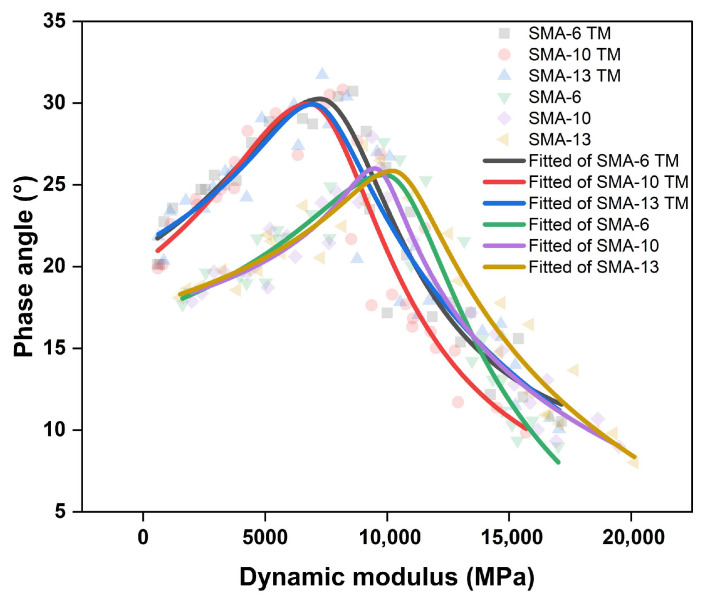
Phase angle dynamic modulus fitting curve.

The fitting parameters and the fitted curves for the dynamic modulus versus phase angle are shown in Figure 12.

The phase angle exhibits a trend of first increasing and then decreasing as the dynamic modulus increases. The phase angles of SMA TM and SMA reach their peaks near E* = 7000 MPa and E* = 11,000 MPa, respectively. The table of fitting parameters shows that the peak of the dynamic modulus–phase angle curve for the semi-dense graded asphalt mixtures is much higher than that for the dense-graded SMA asphalt mixtures. Furthermore, the w-values from the fitted curves for SMA TM are all greater than those for SMA and gradually decrease as the maximum nominal aggregate size increases. When the dynamic modulus is very large, the phase angles of all gradations tend to converge, whereas when the dynamic modulus is smaller, the larger the porosity, and the larger the corresponding phase angle.

From the perspective of energy dissipation, the semi-dense graded asphalt mixtures exhibit a stronger energy dissipation capacity. Their higher peak phase angle implies that, for the same dynamic modulus, the proportion of the loss modulus is larger, resulting in a stronger energy dissipation capacity, which contributes to noise reduction. Furthermore, the larger w-value of the semi-dense graded mixtures indicates that they can effectively dissipate energy over a wider range of dynamic modulus, making them adaptable to a broader spectrum of temperature and frequency conditions.

#### 3.2.3. Macro Analysis of Energy Dissipation Characteristics

**Figure 13:**
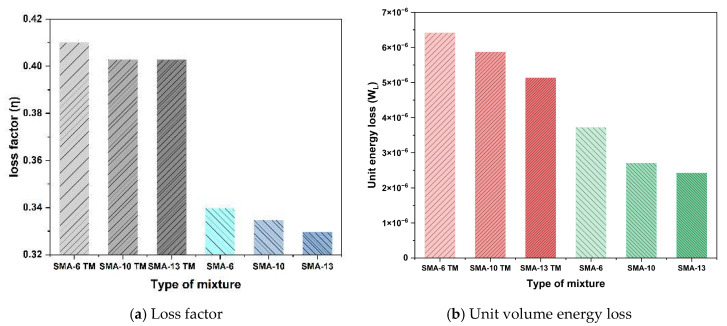
Loss factors and energy losses of different graded mixtures.

Figure 13 presents the energy dissipation and loss factor for the different graded mixtures.

While the loss factor for all mixtures showed a gradual decline with increasing maximum nominal aggregate size, it was consistently higher for the semi-dense graded mixtures compared to their dense-graded counterparts. This trend is also reflected in the energy loss per unit volume, indicating that the semi-dense graded asphalt mixtures can effectively dissipate more vibrational energy, thereby contributing to noise reduction.

**Figure 14:**
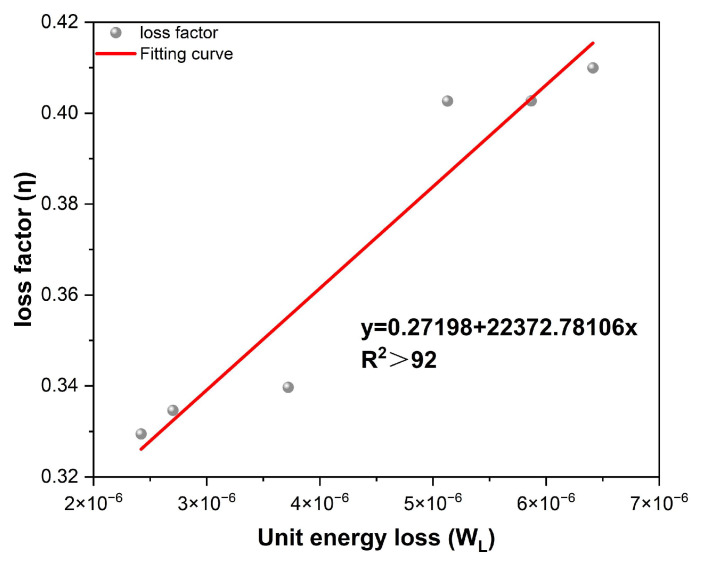
Correlation analysis between loss factor and loss energy.

According to the definitions of the loss factor and dissipated energy, both are positively correlated with the phase angle and dynamic modulus. The relationship between these parameters was analyzed via linear fitting, as depicted in Figure 14.

The loss factor of the mixtures of different gradation types is positively correlated with the energy dissipation per unit volume, with correlation coefficients greater than 0.92. As indicated in the preceding analysis of the damping ratio, under resonance conditions, the value of the damping ratio is half that of the loss factor. Therefore, calculating the loss factor of the mixtures from the dynamic modulus can effectively reflect the actual energy loss and damping ratio characteristics of different asphalt mixtures.

### 3.3. Damping Characteristic Analysis

#### 3.3.1. Damping Ratio

**Figure 15:**
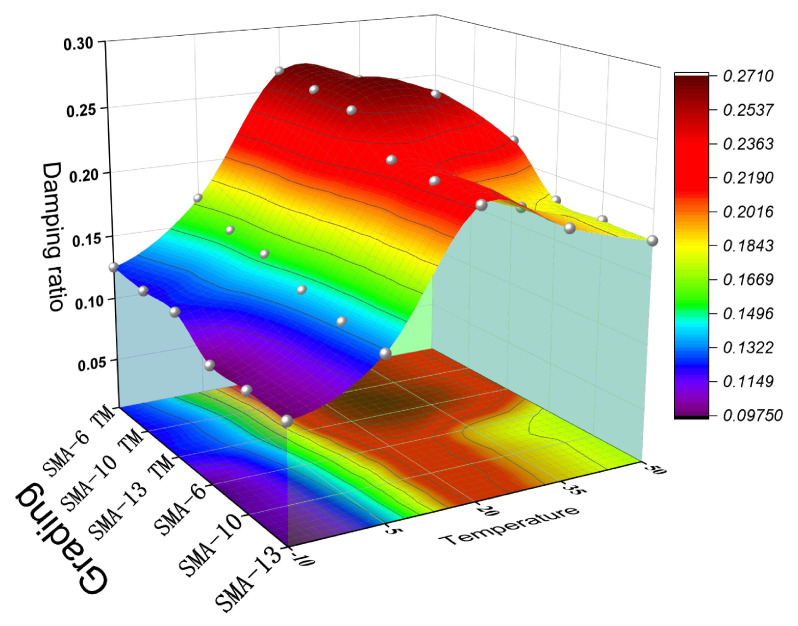
Damping ratio.

Figure 15 shows the variation in the damping ratio for the six different gradations at various temperatures. The damping ratio is a key parameter for measuring the energy dissipation capacity of a material, reflecting the efficiency of energy loss during the vibration process.

As can be seen from the figure, all damping ratios exhibit a trend of first increasing and then decreasing as the temperature rises, reaching a maximum value at 20 °C. Below 20 °C, the damping ratio of the semi-dense graded mixtures is significantly affected by the maximum aggregate size. When the maximum aggregate size is smaller, the damping performance of the material is better, enabling it to absorb and dissipate vibrational energy more effectively. With increasing temperature, the rheological properties of the asphalt material become more pronounced, and the asphalt between the aggregates softens, leading to an increased frequency of relative sliding among the aggregates. During this process, the influence of aggregate size on the damping ratio gradually diminishes. Especially in the high-temperature region, the effect of aggregate size on the damping ratio almost disappears, and the variation in the damping ratio is predominantly governed by the material’s rheological properties. Compared to SMA TM, SMA has a denser structure, which makes the material’s damping ratio at low temperatures less affected by changes in aggregate size. The damping ratio of the semi-dense graded asphalt mixtures is significantly higher than that of the dense-graded asphalt mixtures, which means that the pavement material or structure can more effectively dissipate the vibrational energy generated by vehicle traffic, thereby reducing vibration-induced noise.

#### 3.3.2. Analysis of Energy Amplification Coefficient and Transfer Coefficient

**Figure 16:**
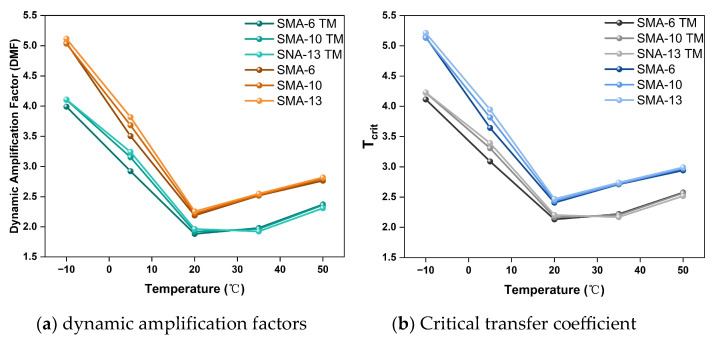
Damping ratio-related parameters.

Among the damping parameters of asphalt mixtures, the maximum dynamic amplification factor, ${DMF}_{max}$, and the critical transmission coefficient, T, are crucial in the process of vibration energy transmission and dissipation; their variations directly affect the material’s noise reduction effect. Figure 16 shows the variation trends of ${DMF}_{max}$ and T for each mixture at different temperatures.

The results indicate that the variation patterns of ${DMF}_{max}$ and T are negatively correlated with the damping ratio. Specifically, ${DMF}_{max}$ and T reflect the system’s ability to amplify external vibration energy and to transmit vibration energy within the material or at its interfaces, respectively. When an external vibration acts on the material, the semi-dense graded structure can significantly reduce the system’s amplification effect on the vibration, thereby enhancing the vibration isolation effect. More of the vibration energy is reflected, absorbed, or dissipated, rather than being effectively transmitted into the material’s interior. This energy conversion process means that most of the vibrational energy is dissipated as thermal energy within the material, which reduces the energy generated by system resonance and also decreases the acoustic waves radiated into the surrounding medium, thereby effectively reducing tire–pavement vibration noise.

### 3.4. Analysis of Noise Reduction Effect

#### 3.4.1. Indoor Damping and Noise Reduction Characteristics

**Figure 17:**
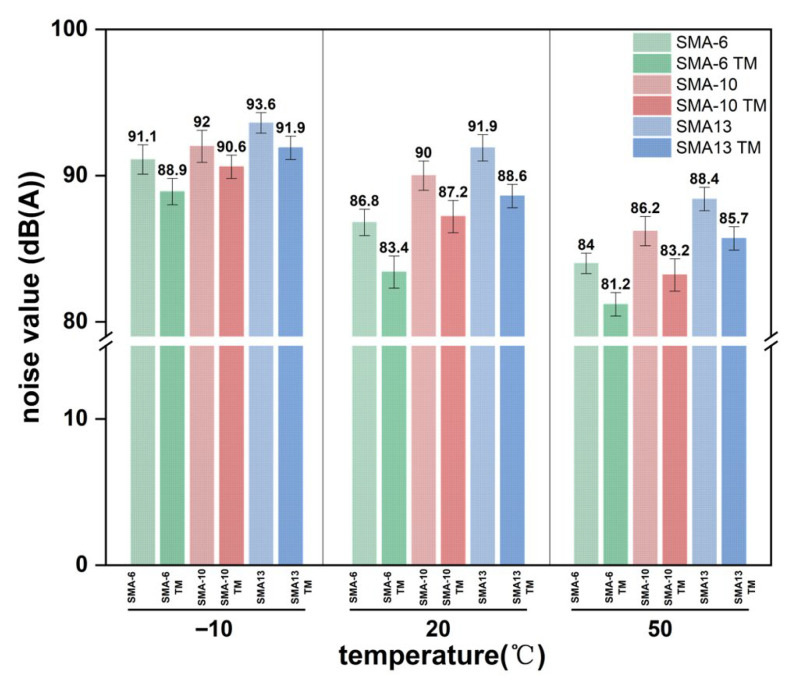
Noise diagram.

Each graded mixture showed relatively small differences in its dynamic modulus and phase angle within the −10 to 5 °C and 35 to 50 °C temperature intervals, while the phase angle reached its peak near 20 °C. Based on this phenomenon, this study conducted a tire drop test to measure pavement noise at −10, 20, and 50 °C, with the tests results shown in Figure 17.

The noise levels of the SMA and SMA TM mixtures decreased as temperature increased and increased as aggregate size increased; among them, the overall noise of SMA TM was consistently lower than that of SMA, demonstrating superior noise reduction performance. Its noise variation trend is highly consistent with the variation pattern of the dynamic modulus with temperature, reflecting a clear correlation between the two. The average noise reduction magnitudes of SMA TM at the different temperatures were 1.78 dB(A), 3.12 dB(A), and 2.83 dB(A), respectively. The noise reduction effect was most significant at 20 °C, indicating that its damping performance is strongest under normal temperature conditions, effectively dissipating vibrational energy. Furthermore, although the damping ratio decreased and energy dissipation was reduced at high temperatures, the noise did not show an increase. This is because the high temperature softens the asphalt, causing the material’s viscoelastic properties to shift towards increased viscosity and fluidity. The magnitude of the stiffness reduction exceeded the effect of the decrease in the phase angle, leading to a lower proportion of the loss modulus. However, due to the reduced friction coefficient and surface stiffness, friction noise and aerodynamic noise decreased simultaneously, thereby keeping the overall noise level low. This result verifies that the energy loss characteristics and noise reduction capability of the material can be effectively predicted using the dynamic modulus and phase angle.

**Figure 18:**
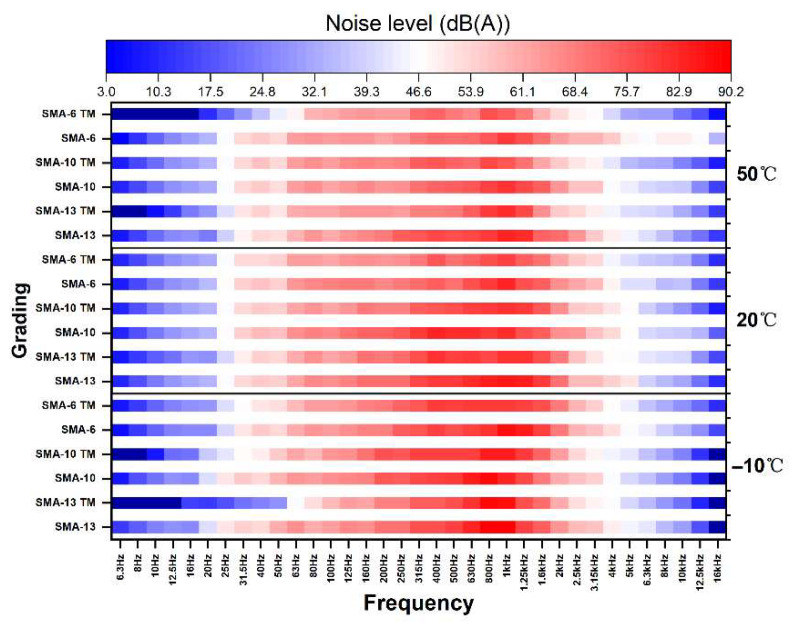
Noise spectrum diagram.

To comprehensively evaluate how the dynamic modulus affects damping-induced vibration and noise reduction, it is insufficient to rely solely on a comparison of noise values, because the noise detection equipment calculates the noise value from the 1/3 octave band noise levels, integrated using A-weighting. Figure 18 shows the spectrogram in the 1/3 octave bands.

The noise level is represented by a color gradient, from low to high: blue, white, and red. The peak of the vibration noise is mainly concentrated around 800 Hz and exhibits a distinct horn effect. Under low-temperature conditions, SMA TM effectively reduces high-frequency and low-frequency noise, and the noise peak gradually decreases as the temperature increases. In medium- and high-temperature environments, there is little difference in the noise reduction effect between the semi-dense and dense-graded mixtures in the low-frequency band, but the high-frequency noise decreases significantly with increasing temperature. Furthermore, the noise reduction effect of the semi-dense graded mixtures is significantly influenced by aggregate size: at low temperatures, the noise peak is lower with smaller aggregate sizes, whereas with larger aggregate sizes, the noise reduction effect for high and low frequencies is better, but the peak frequency noise is higher. Therefore, the overall noise reduction performance is superior with smaller aggregate sizes. The spectral analysis shows that the noise reduced by the damping effect is most prominent within the 500–1250 Hz frequency range. Under medium- and high-temperature conditions, the capacity of SMA-TM to mitigate high-frequency noise remains substantial, and the magnitude of this reduction increases as the temperature rises. This result indicates that the synergistic effect of temperature and aggregate size is a key factor in optimizing the noise reduction strategy for semi-dense graded mixtures.

#### 3.4.2. Evaluation of Noise Reduction Effect on Tests Road

**Figure 19:**
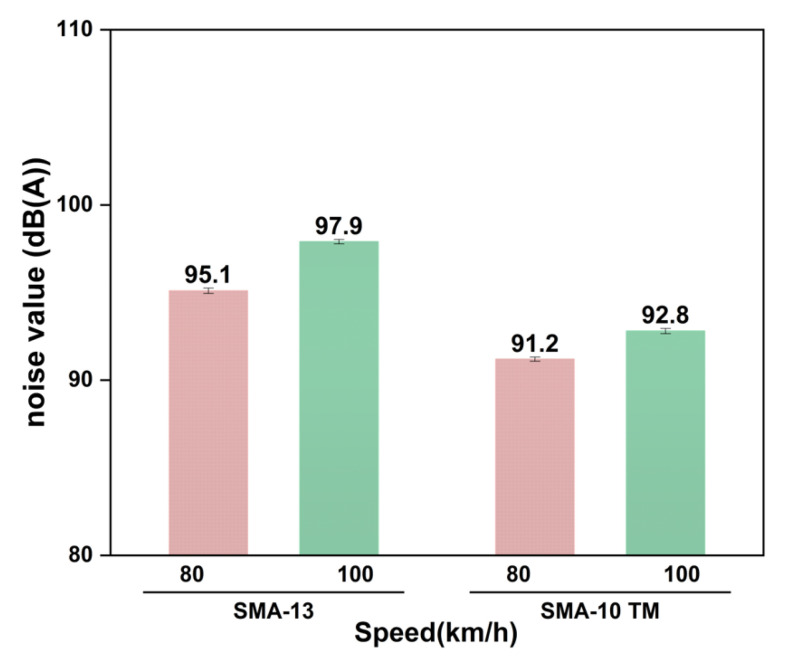
CPX testing noise analysis.

Figure 19 shows the CPX-measured noise values on the two graded test roads at different vehicle speeds.

The tests results show that at vehicle speeds of 80 km/h and 100 km/h, the noise of the SMA-10 TM tests section was reduced by 5.2 dB(A) and 3.9 dB(A), respectively, compared to the SMA-13 tests section, demonstrating a significant noise reduction effect. Furthermore, with an increase in vehicle speed from 80 km/h to 100 km/h, the noise of SMA-13 increased by 2.8 dB(A), whereas the noise of SMA-10 TM increased by only 1.2 dB(A), indicating that the semi-dense gradation has greater noise reduction stability. Therefore, the noise reduction effect of the semi-dense graded asphalt mixture is superior.

Achieving a 3 dB(A) reduction in noise levels has the same perceptual effect as doubling the distance from the sound source, reducing traffic volume by half, or decreasing vehicle speed by 25%, as dictated by acoustic principles [59]. Therefore, paving with semi-dense graded asphalt mixtures can significantly reduce the noise impact on the surrounding environment, making them particularly suitable for expressways in residential areas with high noise reduction requirements. This material not only helps to increase speed limits but also reduces the costs associated with establishing green belts and sound barriers, thus offering significant economic advantages. While its noise reduction benefits are most pronounced at high speeds, its robust overall performance makes it a versatile and reliable solution for noise mitigation across a wide range of road networks.

## 4. Conclusions

To address traffic noise from tire–pavement interaction, this study developed and validated an optimized asphalt mixture, SMA-TM. The core objective was to reduce noise by enhancing the material’s energy dissipation and damping properties while maintaining essential road performance. This was confirmed through systematic laboratory evaluations and on-site CPX tests. The main conclusions are as follows:

Firstly, SMA-TM’s road performance meets design requirements. While its high-temperature stability was approximately 11% lower and its low-temperature performance decreased by 4–14% compared to the dense-graded asphalt mixture, its moisture stability remained excellent (TSR values around 90%), comparable to the conventional SMA. This demonstrates that the optimized gradation design provides SMA-TM with sufficient durability for its intended application.

Secondly, SMA-TM’s core noise reduction mechanism is its superior energy dissipation capacity. Its unit energy loss reached approximately 5.5 × 10^−6^, more than double that of conventional dense-graded asphalt (~2.5 × 10^−6^), enabling it to effectively absorb vibrational energy and thereby reduce noise.

Furthermore, SMA-TM’s damping characteristics are superior to conventional SMA, with its loss factor increasing significantly from 0.33 to 0.41. This allows it to more effectively convert vibrational energy into heat, achieving an average noise reduction of over 3 dB(A) within the critical 500–1250 Hz frequency range.

Finally, on-site CPX tests confirmed SMA-TM’s excellent noise reduction and stability in real-world applications. This effect is particularly pronounced at high speeds: when vehicle speed increased from 80 to 100 km/h, the pavement noise rose by only 1.2 dB(A), demonstrating its superior noise reduction stability.

In summary, through its sophisticated optimized gradation design, SMA-TM not only ensures road performance but also demonstrates excellent noise reduction effects, providing an innovative solution for urban transportation development that combines significant economic and environmental benefits.

## 5. Limitations and Future Work

The proposed low-noise asphalt technology is positioned as a pragmatic and complementary harm-reduction strategy for urban transportation, designed to mitigate the immediate public health impacts of traffic noise during the long-term transition to more sustainable transport systems. Its sustainability value is supported by the use of durable, 100% recyclable asphalt and its future-proof relevance in mitigating tire–pavement noise, which remains the dominant source for electric vehicles. However, realizing this technology’s full potential requires acknowledging the boundaries of the present investigation. The current findings, based on a newly constructed pavement with standard reference tires in a single locale, necessitate future research to assess performance evolution under aging and clogging, the effects of diverse consumer tires, and applicability across varied climatic regions. This defines a clear research roadmap focused on developing predictive life-cycle acoustic models, exploring synergistic effects with other modifiers such as crumb rubber, and conducting extensive field validations. Such endeavors are essential to confirm the technology’s real-world efficacy and facilitate its wider implementation as a reliable tool for improving the urban acoustic environment.

## Figures and Tables

**Table 1:** Main technical indicators of aggregates.

Aggregate Type	Test Items	Unit	Result	Specification
Coarse aggregate	Crushing value	%	12.4	<18
	Los Angeles abrasion loss	%	8.0	<22
	Apparent specific gravity	t/m^3^	2.926	≥2.6
	Water absorption	%	0.63	≤1.0
Fine aggregate	Apparent relative density	t/m^3^	2.728	≥2.6
	Soundness	%	18	>12
	Sand equivalent	%	67	>65
	Methylene blue value	g/kg	1.3	<2.5
Mineral powder	Apparent density	t/m^3^	2.757	≥2.5
	Moisture content	%	0.6	≤1
	Hydrophilicity coefficient	/	0.82	<1
	Plasticity index	%	2.4	<4

**Table 2:** Main technical indicators of asphalt.

Indicator	Result	Requirement	Specification (JTG E20-2011) [45]
Penetration (25 °C, 5 s, 100 g) (0.1 mm)	49.5	40–60	T 0604
Softening point (°C)	97.9	≥60	T 0604
Ductility (5 °C, 5 cm/min) (cm)	49.8	≥20	T 0606
60 °C dynamic viscosity (Pa·s)	507,881.5	≥200,000	T 0605

**Table 3:** Technical indicators of lignin fiber.

Test Parameters	Unit	Test Results	Technical Requirements
Ash content	%	19.4	13~23
pH value	-	8.0	6.5~8.5
Oil absorption rate	%	6.1	5~9
Moisture content	%	3.7	≤5
Mass loss (210 °C, 1 h)	%	2.6, No combustion	≤6, No combustion
Maximum length	mm	3.8	≤6

**Table 4:** Asphalt mixture aggregate–asphalt ratio.

Gradation Types	Target Porosity	Asphalt–Aggregate Ratio
SMA-6	4%	6.32%
SMA-10		6.42%
SMA-13		6.2%
SMA-6 TM	9%	6.47%
SMA-10 TM		6.26%
SMA-13 TM		6.44%

## Data Availability

The original contributions presented in this study are included in the article. Further inquiries can be directed to the corresponding author.
